# A cruel invasion of Ewing's sarcoma of the skull: A rare case report

**DOI:** 10.1016/j.ijscr.2023.108380

**Published:** 2023-06-06

**Authors:** Farzaneh Zare, Niloofar Shahbazi, Navid Faraji, Rasoul Goli, Behnam Mostafaei, Sina Anari

**Affiliations:** a Department of Nursing, School of Nursing and Midwifery, Urmia University of Medical Sciences, Urmia, Iran; b Department of Nursing, School of Nursing and Midwifery, Islamic Azad University Zarand, Kerman, Iran; c Department of Medical-Surgical Nursing, School of Nursing and Midwifery, Urmia University of Medical Sciences, Urmia, Iran; d Department of Nursing, School of Nursing and Midwifery, Islamic Azad University Maragheh Branch, Tabriz, Iran

**Keywords:** Ewing, Sarcoma, Skull, Tumor, Case report

## Abstract

**Introduction and importance:**

Ewing's sarcoma, a highly malignant bone tumor, typically affects the pelvis and long bones of the lower extremities in children and young adults; primary involvement of the skull is rare. Primary Ewing's sarcoma arising from the skull is very rare. In most cases, this disease is fatal, although the prognosis of Ewing sarcoma improves with radiation and chemotherapy after surgery.

**Case presentation:**

This case is about 25-year-old woman who was referred to Omid Hospital in Urmia because of frequent headaches, where a tumor mass was found according to the results of CT scan. Biopsy confirmed small round cell sarcoma as the diagnosis. Chemotherapy was ineffective and tumor growth was unstoppable, causing the patient to die after 3 months.

**Clinical discussion:**

Ewing's sarcoma can affect various parts of the human body, including bone and soft tissue, but rarely the skull. Ewing's sarcoma typically grows extradural and often reaches a very large size before invading the skull or being detected clinically.

**Conclusion:**

In most cases, Ewing's sarcoma is fatal, although the prognosis of this disease improves with radiation and chemotherapy after surgery.

## Highlights

• Ewing's sarcoma occurs most commonly in children and young adults.
• Primary Ewing's sarcoma of the skull bone is rare.
• In most cases, this disease is fatal.

## 1 Introduction

One type of malignancy that forms from a specific type of cell in bone or soft tissue is Ewing sarcoma. These are undifferentiated small circular cell sarcomas that can also form in bone or soft tissue. Swelling and pain near the tumor are the most common signs and symptoms of Ewing sarcoma [1].

Ewing's sarcoma occurs most commonly in children and young adults, with a peak incidence in the second decade of life. It occurs most commonly in the long bones of the extremities (especially femurs) and pelvis [2]. Primary Ewing's sarcoma of the skull bone is rare and accounts for approximately 1 % of all Ewing's sarcomas, and the temporal bone is most commonly affected, followed by the frontal and parietal bones [3]. In most cases, this disease is fatal, although the prognosis of Ewing's sarcoma improves with radiation and chemotherapy after surgery [4].

Considering the unusual location and soft tissue extension, we report a case of primary Ewing's sarcoma of the occipital bone with soft tissue extension. The work has been reported in line with the SCARE 2020 Criteria [5].

## 2 Presentation of case

**Fig. 1:**
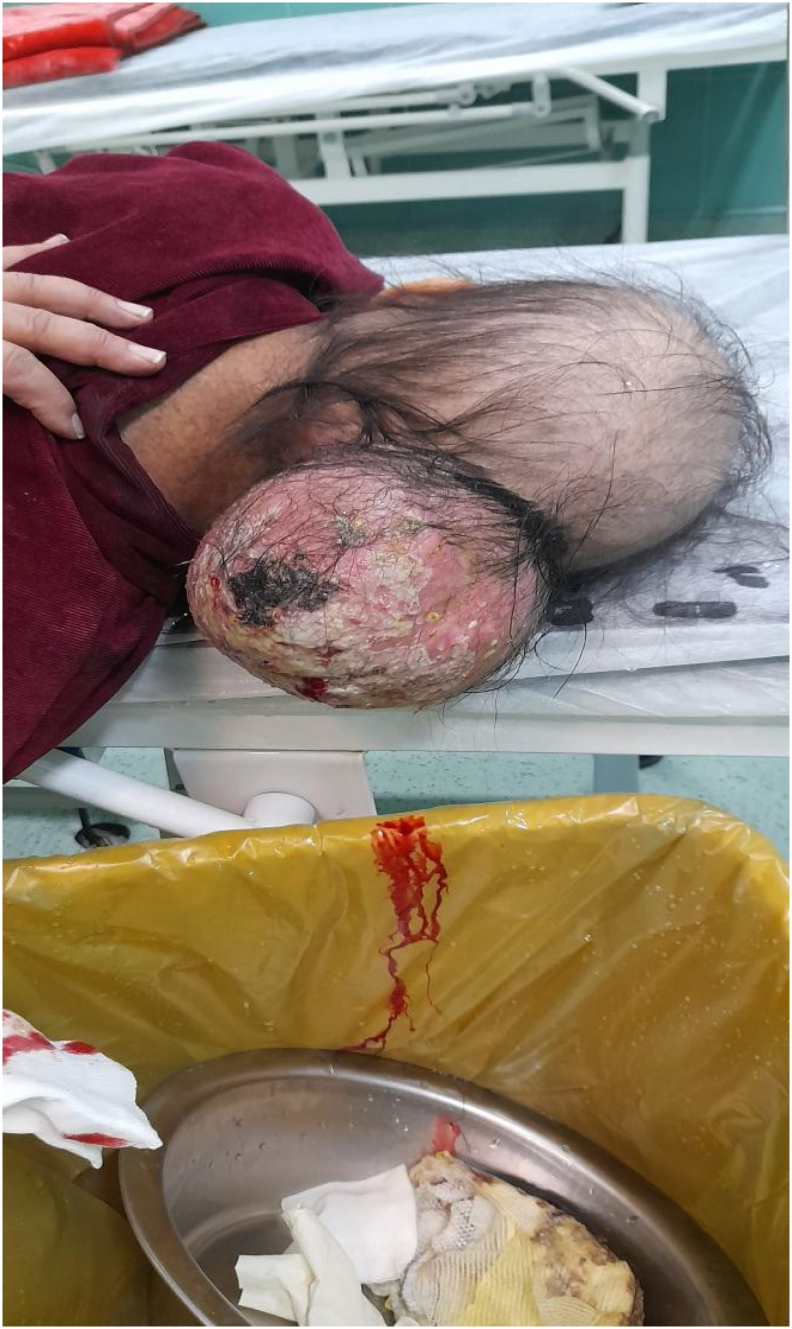
Patient with Ewing's sarcoma.

**Fig. 2:**
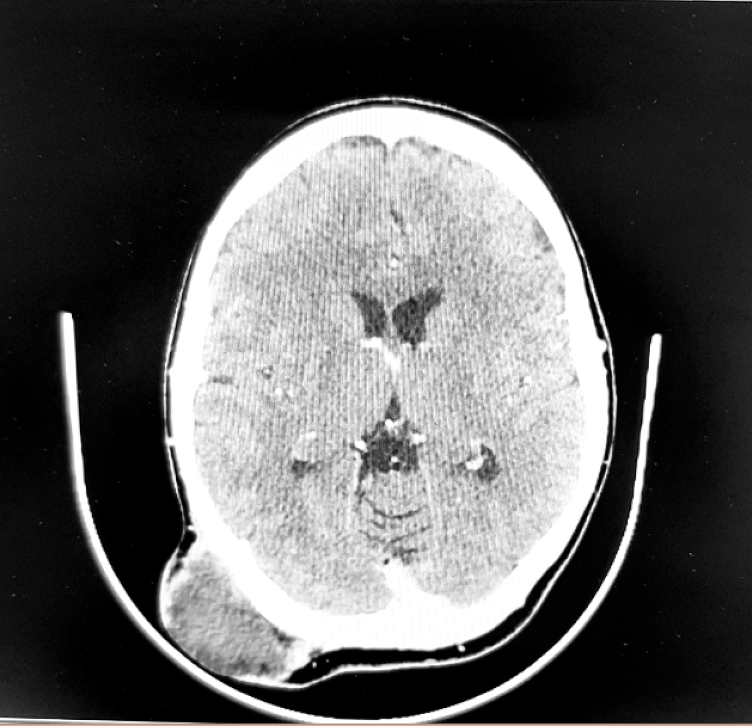
Brain CT scan of patient with Ewing's sarcoma.

**Fig. 3:**
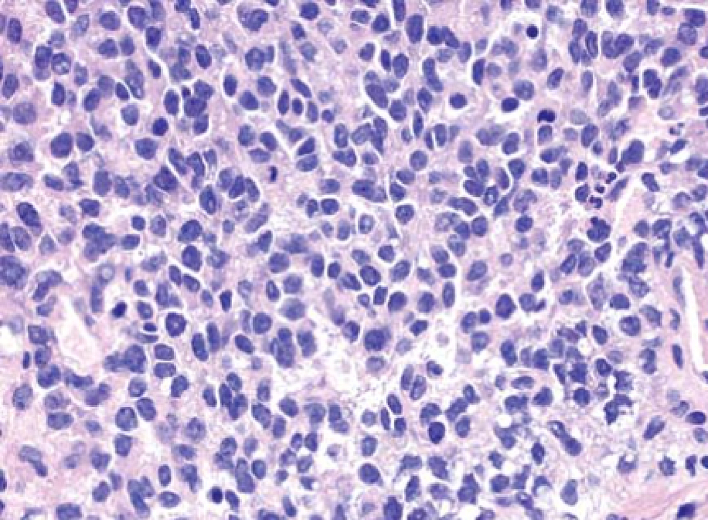
Histological slide of patient with Ewing's sarcoma.

This case report is about a 25-year-old woman who referred to Omid Hospital in Urmia, because of frequent headaches, accompanied by mild nystagmus. After approximately one month, she noticed a protuberance on back of her head. The physical examination revealed tenderness and swelling around the affected area. The patient also exhibited difficulty in range of motion. Additionally, neurological deficits such as weakness or sensory disturbances were slightly presented, including problems in balance. Initial examination by CT revealed the presence of a relatively large mass measuring 5 cm ∗ 5 cm in the occipital region with normal overlying skin (Figs. 1; 2; 3), and it is noteworthy to say that there were no distant metastases in whole body CT control after chemotherapy. According to pre-procedure imaging study (CT) the location of the tumor identified, in the upper distal portion. The patient was ready for biopsy to determine the specific type of tumor by general anesthesia, and the result was a small-cell round cell sarcoma that has gene fusions with a member of the FET gene family, namely EWSR1 (Ewing sarcoma RNA binding protein1). Blood sample examination has shown nothing abnormal.

The size of the tumor increased rapidly which caused dermal complication as desquamation. Dressing changes and dermal cares were additional burden on her shoulder. Overall therapeutic strategy was reduction of the tumor size by chemotherapy to finally it can be removed by surgery, and the specific protocol to treatment was vincristine, doxorubicin, cyclophosphamide and Ifosfamide. The patient received high-dose chemotherapy, but 12 chemotherapy sessions had no effect on shrinking the tumor while the general status of patient including physical and mental has affected tremendously. After chemotherapy, reduction of mature and healthy blood stream cells was obvious on blood sample examination which results in severer fatigue.

Chemotherapy only slowed down the growth rate a little. On the one hand, the patient's physical condition was not able to tolerate further intervention, and on the other hand, the tumor was getting bigger day by day, and no surgery was able to remove the tumor at this size. The patient had balance problems and squinted due to the high pressure in the occipital region. Unfortunately, the patient died after 3 months of struggle.

## 3 Discussion

Bone or soft and connective tissue can be affected by Ewing sarcoma which is most invaded sites by this type of sarcoma. Symptoms include swelling and pain at the tumor site, fever, and bone fractures [6]. The most common sites of origin are the legs, pelvis, and chest wall. The cancer has already scattered to other portions of the body at the time of diagnosis, in about 25 % of cases. Complications may include pleural effusion or paraplegia, meanwhile the main culprit of Ewing sarcoma is idiopathic [7]. Most cases appear to occur incidentally, and sometimes a germline mutation is present. The underlying mechanism often involves a genetic alteration known as a reciprocal translocation [8].

Diagnosis can be done by biopsy of the malignant tumor, and treatment often includes chemotherapy, radiation therapy, surgery and stem cell transplantation. Targeted therapies and immunotherapies are currently being investigated. The five-year survival rate is approximately 70 %. However, this estimate is influenced by a number of factors [9].

Ewing sarcoma is more common in men (1.6 men to 1 woman) and usually occurs in childhood or early adulthood, with a peak between 10 and 20 years of age. It can occur anywhere in the body, but most commonly in the pelvis and proximal long tubular bones, especially near the growth plates. Affected individuals usually suffer from severe bone pain. In rare cases, it can also occur in the vagina. Signs and symptoms include intermittent fever, anemia, leukocytosis, increased erythrocyte sedimentation rate, and other symptoms of inflammatory systemic disease [10].

The most prevalent symptoms are swelling, localized pain, and sporadic bone pain of ranging intensity, based on the Bone Cancer Research Trust (BCRT). Swelling is most likely to be visible if the sarcoma is on a bone near the surface of the body, but if it occurs elsewhere deeper in the body, such as in the pelvis, it may not be visible, however the final diagnosis is based on histomorphology findings, immunohistochemistry, and molecular pathology [11].

Ewing's sarcoma is a small, blue-round cell tumor that typically shows clear cytoplasm on hematoxylin and eosin staining, which is due to glycogen. Other entities with similar clinical presentations include osteomyelitis, osteosarcoma (especially telangiectatic osteosarcoma), and eosinophilic granuloma. Soft tissue neoplasms such as pleomorphic undifferentiated sarcoma [12]. Almost all patients receive chemotherapy with multiple agents (usually vincristine, doxorubicin, cyclophosphamide, Ifosfamide and etoposide) and local disease control by surgery and/or radiation. An aggressive approach is necessary because almost all people with what appears to be localized disease at the time of diagnosis actually have asymptomatic metastatic disease [13].

Although primary involvement of the skull is rare, accounting for approximately 1 % of all Ewing sarcomas, metastases to the skull are not uncommon. The frontal and parietal convexities are relatively commonly involved. Ewing's sarcoma generally grows extradural and often achieves a huge size before dural invasion or clinical finding, or both [14]. Symptoms usually develop as a result of dural invasion, hydrocephalus, or increased intracranial pressure. Headache and swelling of the scalp are the most common symptoms, and papilledema is the most common sign. Our case had a huge tumor size, and as we said increased intracranial pressure caused visual problems [15].

Asifur and et al., in a case report study have explained a rare situation about an 18-year-old right-handed boy with a rapidly enlarging, paramedian swelling in the frontoparietal region for a duration of 3 months [16]. This case has a great similarity with our patient, but the location of the tumor was different.

In another case study, Gupta and et al., reported on an 11-year-old girl who presented with a painless swelling in the left frontoparietal region of the scalp for 8 months that gradually increased in size. There was no history of headache, vomiting, fever, seizures, or other focal neurologic deficits. The outer surface of the soft tissue was smooth and partially encapsulated. This case also has similarity to our case report, but in a smaller size and a longer duration of disease [17].

Patients with this lethal syndrome, even if they survive longer than expected, become severely impaired and palliative care should be considered. The goals of palliative care are to prevent and relieve pain and provide support for families. Such care includes planning with the family about the practicalities of the death and continuing family support after the patient dies [18,19].

## 4 Conclusion

It is noteworthy to be said that Ewing's sarcoma of the skull can have considerable growth, which in most cases is unlimited and difficult to stop. Radiotherapy and chemotherapy can slow down the growth of the tumor, but they cannot solve the problem, so that most patients are affected by an uncontrolled growth of the tumor in a short time, which means the end of their life.

## Consent

Written informed consent was obtained from the patient for publication of this case report and accompanying images. A copy of the written consent is available for review by the Editor-in-Chief of this journal on request.

## Ethical approval

Ethical approval for this study was provided by the Ethics Committee of Urmia University of Medical sciences, West Azerbaijan, Iran on 10 February 2022 (Ethics No. IR.UMSU.REC.1398.434).

## Funding

This CASE REPORT did not receive any specific grant from funding agencies in the public, commercial, or not-for-profit sectors.

## Author contribution

Rasoul Goli, Behnam Mostafaei and Navid Faraji: Study concept, data collection, writing the paper and making the revision of the manuscript following the reviewer's instructions. Sina Anari, Niloofar Shahbazi and Farzaneh Zare: Study concept, reviewing and validating the manuscript's credibility.

## Guarantor

Rasoul Goli.

## Research registration number

Not applicable.

## Provenance and peer review

Not commissioned, externally peer-reviewed.

## Declaration of competing interest

None.
